# A dual transcript-discovery approach to improve the delimitation of gene features from RNA-seq data in the chicken model

**DOI:** 10.1242/bio.028498

**Published:** 2017-11-28

**Authors:** Mickael Orgeur, Marvin Martens, Stefan T. Börno, Bernd Timmermann, Delphine Duprez, Sigmar Stricker

**Affiliations:** 1 Freie Universität Berlin, Institut für Chemie und Biochemie, Thielallee 63, 14195 Berlin, Germany; 2 Max Planck Institute for Molecular Genetics, Development and Disease Group, Ihnestrasse 63-73, 14195 Berlin, Germany; 3 Sorbonne Universités, UPMC Univ. Paris 06, CNRS UMR 7622, Inserm U1156, IBPS-Developmental Biology Laboratory, 9 Quai Saint-Bernard, 75252 Paris Cedex 05, France

**Keywords:** Chicken genome annotation, *Gallus gallus*, Gene prediction, Genome-guided transcript discovery, RNA sequencing, Transcriptome reconstruction

## Abstract

The sequence of the chicken genome, like several other draft genome sequences, is presently not fully covered. Gaps, contigs assigned with low confidence and uncharacterized chromosomes result in gene fragmentation and imprecise gene annotation. Transcript abundance estimation from RNA sequencing (RNA-seq) data relies on read quality, library complexity and expression normalization. In addition, the quality of the genome sequence used to map sequencing reads, and the gene annotation that defines gene features, must also be taken into account. A partially covered genome sequence causes the loss of sequencing reads from the mapping step, while an inaccurate definition of gene features induces imprecise read counts from the assignment step. Both steps can significantly bias interpretation of RNA-seq data. Here, we describe a dual transcript-discovery approach combining a genome-guided gene prediction and a *de novo* transcriptome assembly. This dual approach enabled us to increase the assignment rate of RNA-seq data by nearly 20% as compared to when using only the chicken reference annotation, contributing therefore to a more accurate estimation of transcript abundance. More generally, this strategy could be applied to any organism with partial genome sequence and/or lacking a manually-curated reference annotation in order to improve the accuracy of gene expression studies.

## Abstract (precis)

**Summary:** We combined genome-guided gene prediction and whole transcriptome assembly from RNA sequencing data to improve the chicken genome annotation. This method may be also applicable to other imperfectly annotated genomes.

## INTRODUCTION

Since its first release in 2004 and despite significant improvements over the last past decade, the *Gallus gallus* genome is presently incomplete and highly fragmented (Hillier et al., 2004). The chicken karyotype is composed of 38 autosomal chromosomes (1-38) and two additional sex chromosomes (W, Z) (Bloom et al., 1993). Out of these autosomal chromosomes, 10 are macrochromosomes (1-10), with lengths similar to those in mammals, and 28 are microchromosomes (11-38), with lengths ranging from 2 to 25 Mb (Hillier et al., 2004). Chicken microchromosomes display a high recombination rate, contain an elevated number of repetitive elements and are GC-rich, which induces significant bias and sequencing errors when using high-throughput technologies (Chen et al., 2013; Dohm et al., 2008). In addition, microchromosomes are gene dense and enriched in CpG islands, which is the result of short intronic sequences (McQueen et al., 1998; Smith et al., 2000). The fourth version of the *Gallus gallus* genome (galGal4), released in November 2011, has not fully overcome these issues. Out of the 40 chromosomes, 31 are sequenced (1-28, 32, W, Z) and contain more than 9000 gaps, while nine chromosomes remain missing (29-31, 33-38). The genome is also composed of ∼16,000 additional contigs that are not assigned to any chromosome or assigned with low confidence. In total, the galGal4 genome sequence has a size of 1.05 Gb.

RNA sequencing (RNA-seq) data processing and results are highly dependent on the quality of the genome sequence and the associated gene annotation model. Read mapping is one of the critical steps that will further influence sample normalization, gene expression quantification and the identification of relevant genes. Gene expression profiles rely on the alignment of RNA-seq reads along the available reference genome or transcriptome, followed by their assignment to gene features. An incomplete genome sequence coupled with an inaccurate definition of gene features induce a bias in the gene expression quantification and transcript abundance estimation (Jiang and Wong, 2009; Trapnell et al., 2010). Whole transcriptome sequencing offers valuable resources to detect novel genes and transcripts as well as to identify alternative splicing variants (Denoeud et al., 2008; Wang et al., 2008). Depending on the context, two main strategies are widely used to analyze RNA-seq data (Garber et al., 2011). One approach consists of the mapping of reads along the reference genome followed by gene prediction (Guttman et al., 2010; Trapnell et al., 2010; Yassour et al., 2009). This method can be combined with an existing reference annotation in order to detect new transcripts with respect to the provided gene annotation model (Roberts et al., 2011). The second approach aims at reconstructing the whole transcriptome independently of the reference genome (Birol et al., 2009; Grabherr et al., 2011; Robertson et al., 2010). This method is particularly suitable to study models with partial or missing genome sequence. The choice between these approaches greatly depends on the biological question and whether a reference genome is available (Conesa et al., 2016).

When analyzing RNA-seq data obtained from chick embryonic limb cell cultures (so-called micromass cultures) by using the galGal4 reference genome and annotation, we observed that only 62.2% of sequencing read pairs were assigned to gene features, while 86.7% of the read pairs were mapped against the genome sequence. By comparison with the human genome, which has been nearly completely sequenced and accurately annotated, a similar analysis of RNA-seq data obtained from human blood samples depicted an assignment rate to gene features of 81.8% with a mapping rate of 92.3% (Zhao et al., 2015). We hypothesized that information was lost during the analysis of chick RNA-seq data: (i) at the mapping step, either due to low-quality sequencing reads or to missing genome sequence; and (ii) at the read assignment to gene features, which can be due to missing or partially annotated transcripts. To address both issues, we performed a dual transcript-discovery approach by means of genome-guided gene prediction and *de novo* transcriptome assembly. The approach described here enabled us to increase the assignment rate of RNA-seq data by nearly 20% as compared to when using the chicken reference annotation, thus contributing to a more robust quantification of gene expression profiles.

## RESULTS

**Fig. 1.**
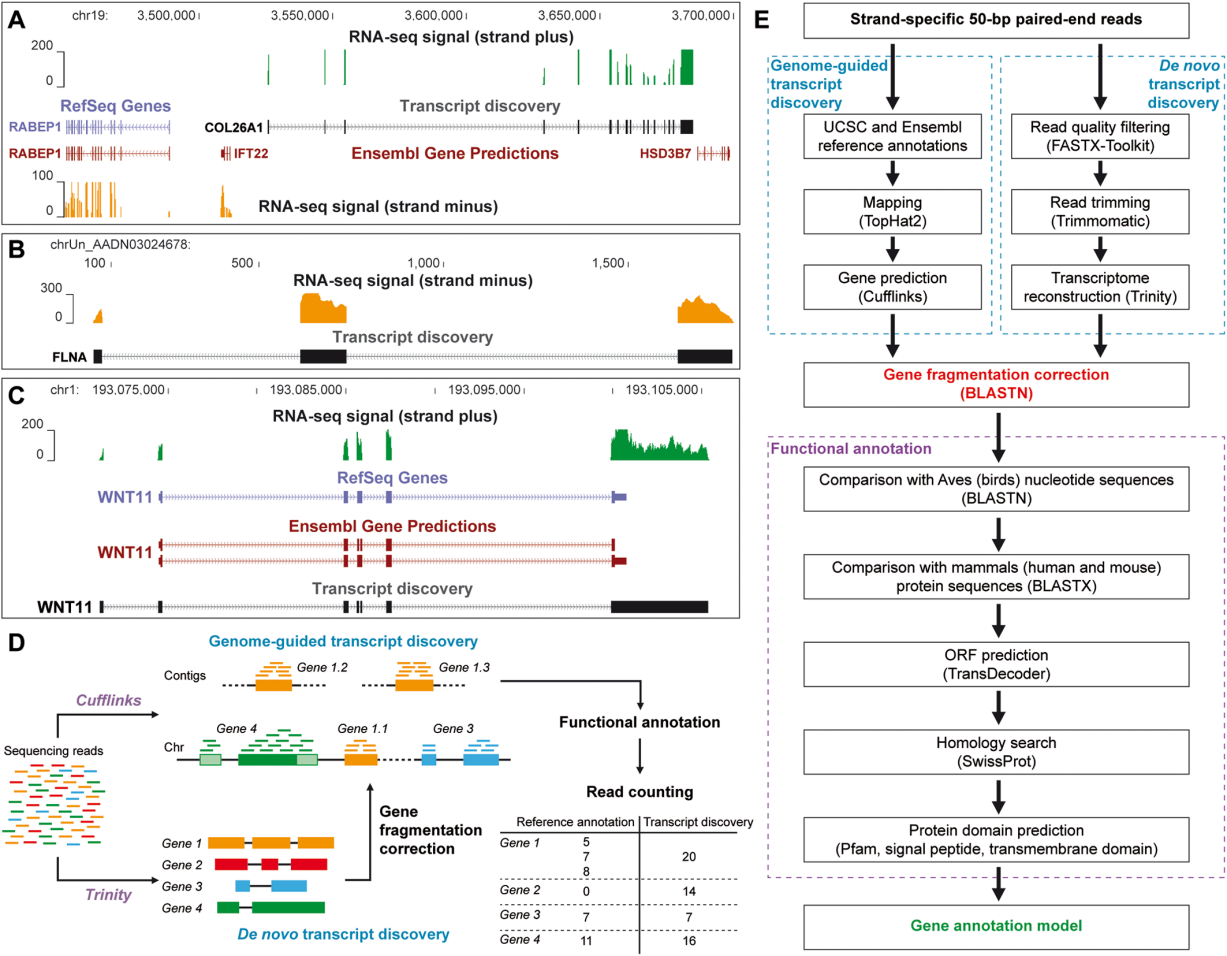
**Dual transcript-discovery approach.** (A) Region surrounding the genes *RABEP1* and *HSD3B7* on chromosome 19. RNA-seq signal on strand plus (green), which does not overlap any gene from UCSC and Ensembl reference annotations, corresponds to the gene *COL26A1*. (B) RNA-seq signal (orange) on strand minus of an uncharacterized contig delimitating three exons of the gene *FLNA*. (C) Region of the gene *WNT11* on chromosome 1. As visible from the RNA-seq signal on strand plus (green), both UCSC and Ensembl reference annotations lack an exon of the 5′-UTR and display a shorter 3′-UTR. (D) The dual transcript-discovery approach combined a genome-guided gene prediction with a *de novo* transcriptome reconstruction. This dual approach enabled us to correct for gene fragmentation (orange), to identify missing gene candidates (red) and to adjust or validate existing annotated genes (green, blue) thus improving the assignment rate of RNA-seq read pairs. (E) Workflow to design the comprehensive gene annotation model.

We performed RNA-seq of two independent biological replicates of chick micromass cultures infected for 5 days with empty RCAS-BP(A) replication-competent retroviral particles. We generated 61.3 and 70.3 million strand-specific read pairs and mapped them against the galGal4 version of the chicken genome by using TopHat2 (Kim et al., 2013) (Table 1). Read assignment was performed by using featureCounts (Liao et al., 2014) and a gene annotation model composed of 17,318 genes resulting from the combination of both UCSC and Ensembl reference annotations that were available at the time of analysis. Surprisingly, while 86.7% of read pairs were mapped against the chicken genome, only 62.2% of read pairs were assigned to gene features (Table 1). Therefore, 28.3% of mapped read pairs were not counted, including 93.7% of these read pairs that were not overlapping with any gene feature (Table 1). Close investigation of these unassigned read pairs highlighted genes that seemed to be absent or partially covered by the UCSC and Ensembl reference annotations (Fig. 1A,B), as well as transcripts with missing or partial exon features (Fig. 1C).

**Table 1.**
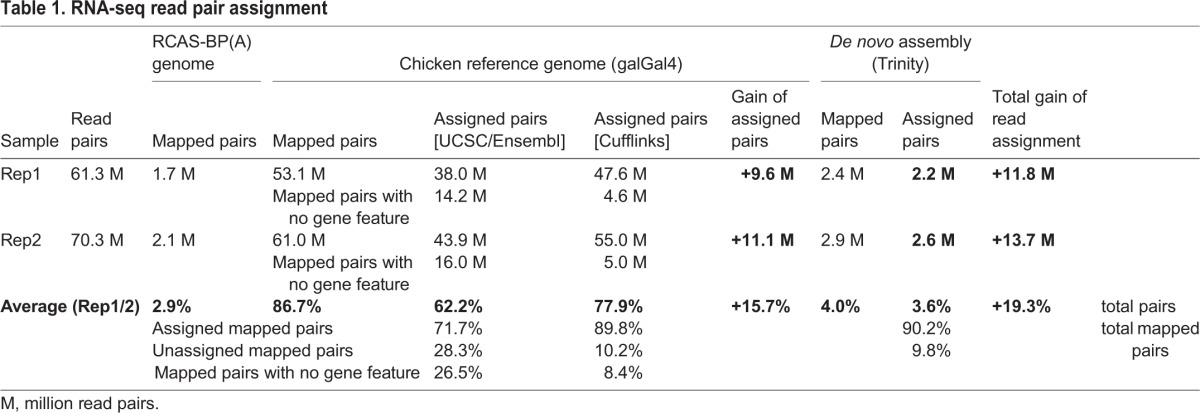
**RNA-seq read pair assignment**

**Fig. 2.**
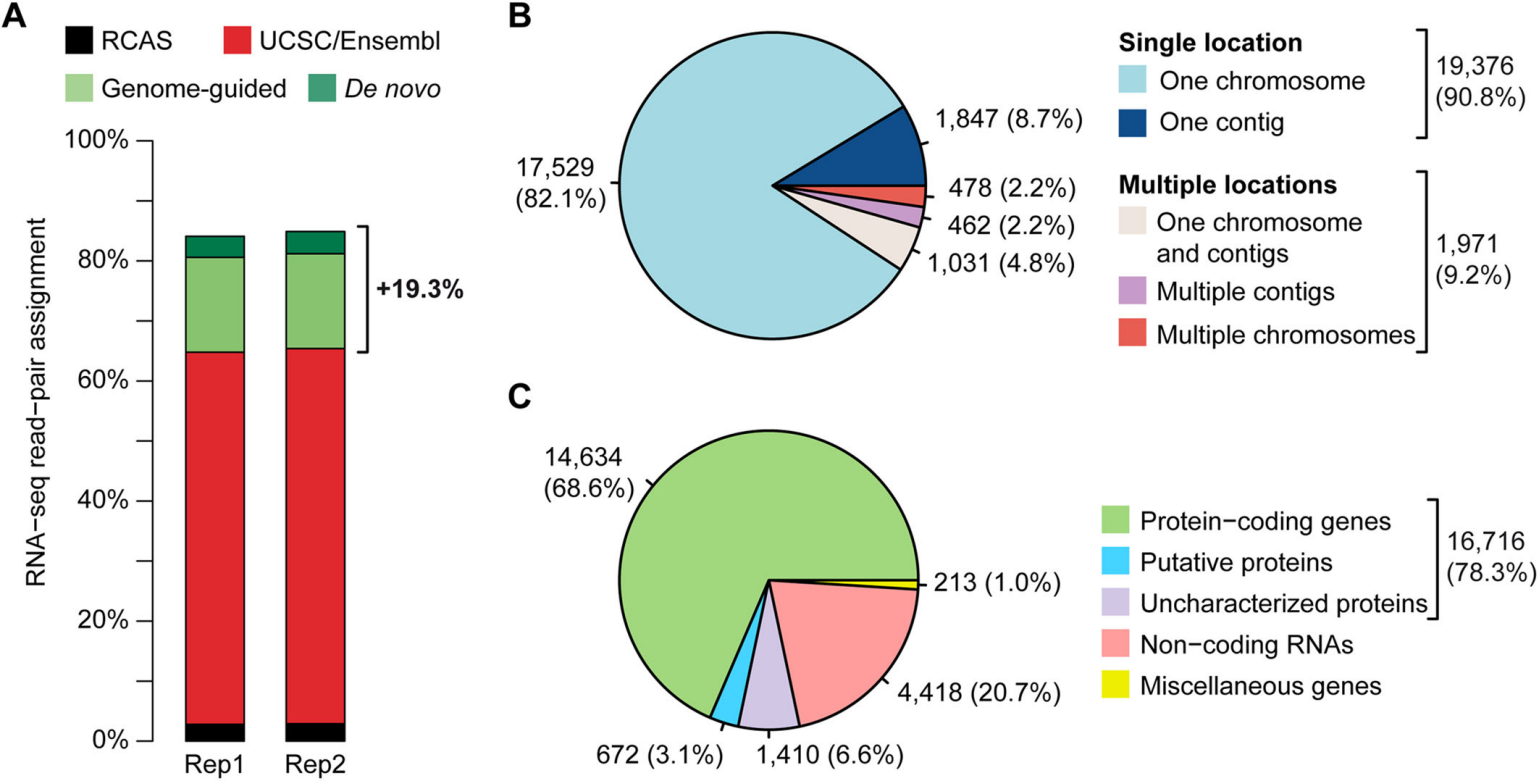
**Characteristics of the new gene annotation model.** (A) The dual transcript-discovery approach combining genome-guided gene prediction (light green) and *de novo* transcriptome reconstruction (dark green) raised the read-pair assignment rate by 19.3% as compared to when using the UCSC and Ensembl reference annotations (red). The proportion of read pairs coming from the RCAS-BP(A) replication competent retroviruses is depicted in black. (B) Proportion of gene locations on chromosomes and contigs of the chicken reference genome galGal4. Of the identified gene candidates, 9.2% are fragmented due to their location on multiple chromosomes and contigs. (C) Proportion of annotated gene biotypes. Most of the annotated gene candidates potentially encode proteins (78.3%). Putative proteins correspond to gene candidates for which at least one protein domain could be detected (3.1%). Uncharacterized proteins are gene candidates with an ORF of ≥100 amino acids without protein domain identified (6.6%). Gene candidates with no sufficient predicted ORF (<100 amino acids) are classified as non-coding RNAs (20.7%). Gene candidates encoding spliceosome complex members and ribosomal RNAs, as well as pseudogenes, are classified as miscellaneous genes (1.0%).

In order to improve the read assignment rate, we first performed a genome-guided transcript discovery by using Cufflinks (Trapnell et al., 2010). This approach was intended to determine more accurately exon-intron junctions, to correct or to complete existing annotated genes, and to identify unannotated gene candidates from the UCSC/Ensembl gene annotation model (Fig. 1D,E). Following this approach, 77.9% of the sequencing read pairs were assigned to gene features, corresponding to 89.8% of the read pairs that were mapped against the genome (Table 1). Therefore, the genome-guided transcript discovery enabled us to raise the read assignment rate by 15.7% as compared to when using both UCSC and Ensembl reference annotations (Table 1). In contrast to genome-guided transcript prediction, *de novo* transcriptome reconstruction relies on overlaps between the sequencing reads to build consensus transcripts, independently of the genome sequence. We therefore applied a genome-independent strategy by using Trinity (Grabherr et al., 2011), in combination with the genome-guided approach, in order to detect transcripts or regions that were not recovered from the genome sequence, such as those located within gaps or uncharacterized chromosomes (Fig. 1D,E). Reconstructed transcripts thus generated were then compared to the gene candidates obtained with the genome-guided approach in order to remove redundant sequences. Full-length transcripts or transcript regions of at least 400 bp that were not assigned to any gene candidate were extracted and grouped as an artificial chromosome. We found that 4.0% of read pairs were mapped against this additional chromosome and 90.2% of these mapped read pairs were assigned to gene features (Table 1). By considering both transcript-discovery approaches, 90.7% of the total read pairs were mapped against the galGal4 chicken genome (86.7%) and reconstructed chromosome (4.0%) (Table 1), and 77.9% and 3.6% of the read pairs were assigned to gene features from the genome-guided and *de novo* transcript-discovery approaches, respectively (Fig. 2A, Table 1). Therefore, 81.5% of the read pairs were assigned to gene features by using this newly established gene annotation model. Given that 62.2% of the sequencing read pairs were assigned to gene features by using both UCSC and Ensembl reference annotations, our transcript reconstruction model enabled us to assign 19.3% more read pairs to gene features (Fig. 2A, Table 1).

The genome-independent transcript assembly also enabled us to correct for gene fragmentation by gathering gene regions located on multiple chromosomes and contigs together (Fig. 1D,E). In contrast to genome-guided transcript discovery, *de novo* reconstruction of transcripts was not limited by the quality of the reference genome sequence. By comparing transcripts generated from both reconstruction approaches, we were able to group dispersed gene features belonging to a same gene candidate together. Although 19,376 (90.8%) gene candidates were found exclusively on a single chromosome or unplaced contig, 1971 (9.2%) gene candidates were identified as being fragmented (Fig. 2B). These fragmented gene candidates included 478 (2.2%) gene candidates that were located on multiple ordered chromosomes, 462 (2.2%) gene candidates split among multiple unplaced contigs, and 1031 (4.8%) gene candidates with regions located on an ordered chromosome and additional unplaced contigs (Fig. 2B).

Transcript prediction and reconstruction approaches did not provide any information on gene name and function. Therefore, gene candidates identified by the dual transcript-discovery approach were then annotated by database comparison and protein domain prediction (Fig. 1E). Gene candidates were first compared to bird gene sequences, taking advantage of the recent increase of available genomic data within avian species and their high DNA sequence conservation (Dalloul et al., 2010; Huang et al., 2013; Jarvis et al., 2014; Schmid et al., 2015; Shapiro et al., 2013; Warren et al., 2010; Zhan et al., 2013; Zhang et al., 2014). Undefined gene candidates were then compared at the protein level to mouse and human databases. Finally, prediction of open reading frames (ORFs) and protein domains was performed on remaining unannotated gene candidates by using homology search against SwissProt and Pfam databases, and sequence analysis tools to identify transmembrane domains and signal peptides. Overall, the computed gene annotation model was mostly constituted of protein-coding gene candidates (16,716, 78.3%) (Fig. 2C). However, 672 (3.1%) gene candidates were only partly annotated (putative proteins having at least one protein domain detected), while 1410 (6.6%) gene candidates remained unannotated (uncharacterized proteins with no protein domain identified but an ORF of at least 100 amino acids). Remaining gene candidates corresponded to miscellaneous genes (213, 1.0%; such as spliceosome complex members, ribosomal RNAs and pseudogenes) and non-coding RNAs (ncRNAs; 4418, 20.7%) for which no sufficient ORF could be predicted (Fig. 2C).

The resulting gene annotation model was composed of 21,347 unique gene candidates, encompassing 5989 additional gene candidates as compared to the UCSC and Ensembl reference annotations associated with the galGal4 genome version. We then compared our results with the most recent version of the chicken genome (galGal5), released in December 2015, which includes 200 additional Mb, three previously missing chromosomes (30, 31, 33) and 23,400 unplaced contigs (Warren et al., 2017). Firstly, strand-specific read pairs were mapped against the galGal5 genome version by using TopHat2 (Kim et al., 2013), and assigned to gene features by using featureCounts (Liao et al., 2014) according to a gene annotation model combining both UCSC and Ensembl annotations. This gene annotation model contained 6280 additional genes as compared to the galGal4 UCSC/Ensembl annotations. Surprisingly, we did not observe any significant improvement of read pair mapping (+1.5%) and assignment (−0.9%) rates despite the increased genome size (Table 2). This indicated that when using galGal5, similar issues will be encountered as with galGal4. Indeed, a comparable number of reads pairs (25.5%) was not associated with any gene feature when mapped against galGal5 (Table 2). Secondly, we compared the predicted gene candidates from our annotation model to the RefSeq annotated galGal5 transcripts. We found that only 52.7% of gene candidates were covered by at least 50% of their total length by galGal5 reference genes (Table 3). In addition, 3958 (18.5%) gene candidates were not detected at all in galGal5 reference genes (Table 3), and 3151 (79.6%) of these corresponded to gene candidates absent from galGal4 UCSC/Ensembl annotations. Lastly, we compared the gene names assigned to gene candidates with galGal5 reference genes that matched at least 50% of their length. Out of the 15,358 gene candidates that were identified in the galGal4 UCSC/Ensembl annotations, 74.1% had a concordant gene name, while 17.9% did not significantly match any galGal5 reference gene (Table 4). Regarding the 5989 additional gene candidates, most of these were not significantly detected among galGal5 reference genes (76.8%) or matched an undefined gene (12.7%) (Table 4). However, 223 (1.0%) gene candidates remaining partly annotated with the dual transcript-discovery approach could be successfully assigned (Table 4).

**Table 2.**
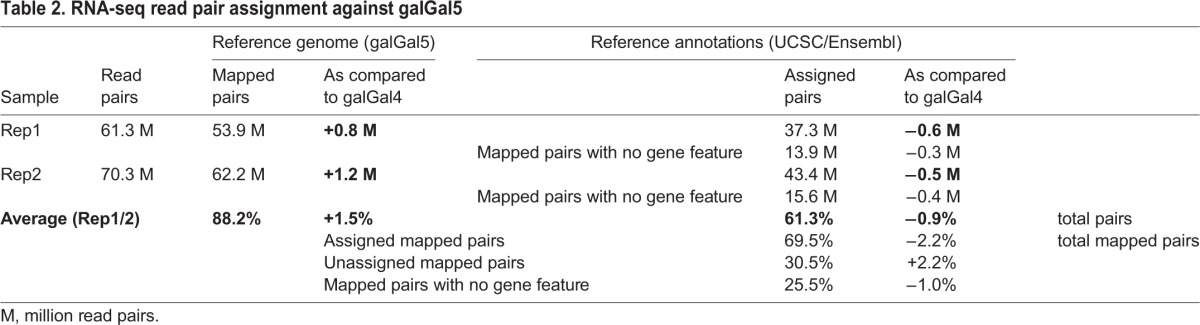
**RNA-seq read pair assignment against galGal5**

**Table 3.**
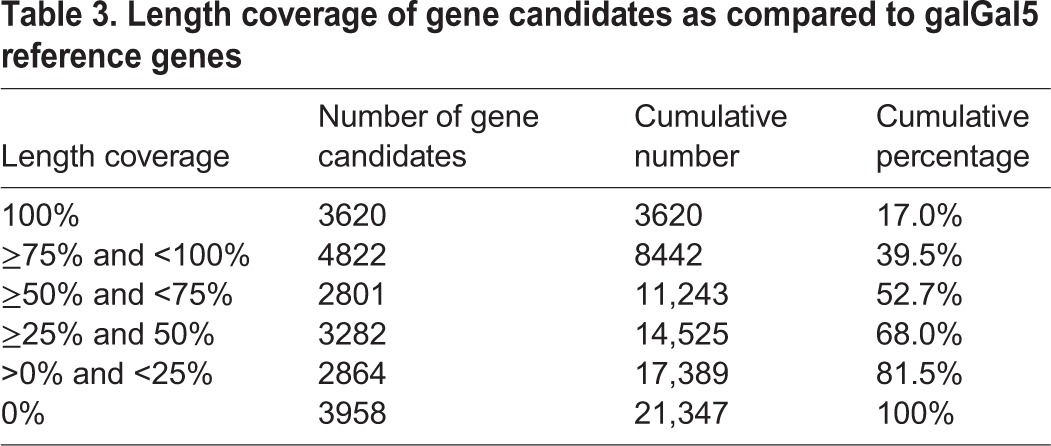
**Length coverage of gene candidates as compared to galGal5 reference genes**

**Table 4.**
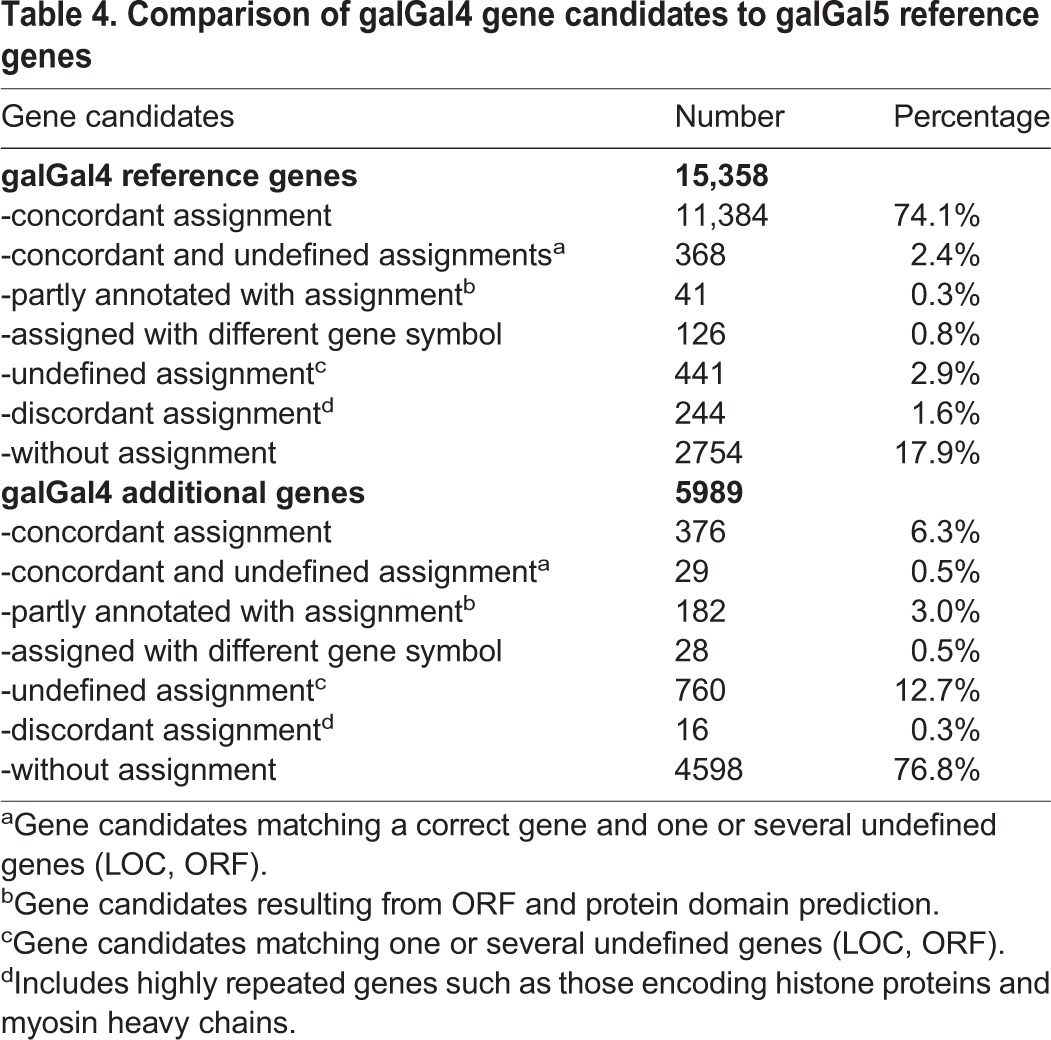
**Comparison of galGal4 gene candidates to galGal5 reference genes**

Altogether, this dual transcript-discovery approach enabled us to define an annotation model of 21,347 gene candidates that includes additional genes as compared to the reference annotation of the chicken genome. Most importantly, it enabled us to retrieve 19.3% more information from the RNA-seq data.

## DISCUSSION

The work presented here describes a dual transcript-discovery approach combining genome-guided gene prediction and *de novo* transcriptome reconstruction, which was applied to improve the assignment rate of RNA-seq data obtained from chicken samples. For the first approach, sequencing read pairs are mapped along the genome followed by a genome-dependent transcript discovery, which computes read coverage and exon-intron junctions from gapped alignments, and distance between both reads of each pair. By contrast, the second approach is carried out independently of the reference genome. Sequencing reads are *de novo* assembled by relying on their overlaps to reconstruct full-length transcripts. Genome-guided transcript discovery is more sensitive than *de novo* transcript reconstruction, but requires a reference genome along which RNA-seq reads are mapped for gene prediction (Garber et al., 2011; Roberts et al., 2011). Therefore, the choice of the latter method is obvious when no or incomplete genome sequence is available. In the case of the chicken model with its partial and fragmented genome sequence, the choice of a complementary transcript-discovery approach, combining both genome-guided and -independent methods, appears suitable to improve RNA-seq data quantification and analysis. While the genome-guided approach contributes to correct existing annotated genes and to identify novel gene candidates, the *de novo* transcript reconstruction compensates for gene fragmentation by associating gene parts located on multiple chromosomes or contigs together; and it identifies gene regions or complete gene candidates that do not belong to the genome sequence due to the presence of gaps or uncharacterized fragments. The new annotation model is composed of 21,347 gene candidates, accounting for 5989 additional gene candidates as compared to the UCSC and Ensembl reference annotations associated with the galGal4 genome version. Of these gene candidates, 1971 (9.2%) have parts spread on multiple locations, while 3340 (15.6%) are identified among the 16,000 unplaced contigs that are not assigned to any ordered chromosome. In addition, the resulting gene annotation model increased the assignment rate of RNA-seq read pairs by 19.3% as compared to when using both galGal4 reference annotations (UCSC and Ensembl), thus contributing to a more accurate estimation of transcript abundance.

It is noteworthy to take into consideration that *de novo* assembly of short reads is prone to cause artefacts and to generate false chimeric transcripts (Yang and Smith, 2013). Such errors can be corrected for instance by comparing reconstructed transcripts with transcripts/proteins of the same organism, closely related organisms, or more accurately annotated organisms. In addition, transcriptome assemblers tend to create multiple transcript sequences per gene, which would cause reads to map at multiple locations and be subsequently ignored during read counting. Several programs have been developed in order to cluster transcript sequences into genes and to remove redundancy. TGICL (Pertea et al., 2003) and CD-HIT-EST (Fu et al., 2012), which were originally designed for clustering of expressed sequence tags (EST), can be used to create consensus gene sequences. However, since both programs perform their clustering based on all transcript sequences, paralogous genes may be erroneously merged. In contrast, Corset (Davidson and Oshlack, 2014) identifies sequence similarity between transcripts by identifying multi-mapped reads resulting from re-mapping of reads against the reconstructed transcriptome. Although this program accurately clusters transcripts into genes, it falls short of building consensus genes from transcript sequences. To overcome these limitations, we applied a strategy that consists in a pairwise comparison of transcript sequences belonging to the same gene candidates followed by incremental concatenation of identical and unique transcript sequences to build full-length gene candidates. Very recently, a similar approach has been reported under the name of superTranscripts (Davidson et al., 2017). We observed that 99.95% of consensus gene sequences generated by superTranscripts were identical to our results. However, we note that superTranscripts tends to remove sequences specific to a unique transcript that do not overlap with any other transcript sequences although being indicated as belonging to the same gene candidates.

Approaches combining genome-dependent and -independent gene prediction have already been proposed before and reported to better recover the transcriptome of a given organism (Davidson et al., 2017; Jain et al., 2013; Visser et al., 2015). However, the approach presented here also includes a method to assign a putative name or function to the gene candidates resulting from gene prediction, which helps with the identification of relevant target genes in downstream analysis. The recent genome sequencing of the zebra finch (Warren et al., 2010), the turkey (Dalloul et al., 2010), the pigeon (Shapiro et al., 2013), the falcon (Zhan et al., 2013), the duck (Huang et al., 2013), and a wide range of additional avian species (Jarvis et al., 2014; Zhang et al., 2014) have provided extensive insights into evolutionary and adaptive traits within birds. DNA conservation of protein-coding genes among avian species considerably facilitated the annotation of the 21,347 gene candidates identified by the dual transcript-discovery approach. By combining DNA sequence comparison against avian genes with protein sequence comparison against mammal species and protein domain prediction, 14,847 (69.6%) gene candidates could be assigned and 672 (3.1%) putative protein-coding gene candidates could be identified. The 5828 (27.3%) remaining gene candidates were divided between uncharacterized proteins and ncRNAs depending on the length of the predicted ORF. However, gene candidates encoding uncharacterized proteins could be also potentially non-coding since none of the protein domains investigated was detected within their putative ORF. On the other hand, ncRNAs remain challenging to annotate according to a recent study comparing an extensive repertoire of long multi-exonic ncRNAs across 11 tetrapods separated by up to 370 million years (Necsulea et al., 2014). Besides their overall weak conservation as compared to protein-coding sequences, long ncRNAs display high tissue specificity and rapidly diverge through evolution, which renders their annotation difficult by comparing with other species.

Since the first draft released in 2004, considerable efforts have been made to improve the *Gallus gallus* reference genome and its annotation (Hillier et al., 2004; Kuo et al., 2017; Schmid et al., 2015; Thomas et al., 2014; Warren et al., 2017). In December 2015, the fifth version of the chicken genome (galGal5) was released (Warren et al., 2017). As compared to the fourth version, this release is 200 Mb longer and includes three additional chromosomes (30, 31, 33) but remains highly fragmented. Indeed, this fifth version is still composed of 15,400 unassigned contigs and 8000 contigs assigned with low confidence, accounting for ∼17% of the total genome size. While we found that some gene candidates still remain missing or partly annotated in this new release, our gene prediction is consistent with other comparisons identifying novel genes absent from galGal4 reference annotation but present in galGal5 reference annotation or other birds (Bornelöv et al., 2017; Hron et al., 2015; Lovell et al., 2014; Warren et al., 2017). Improvement of the chicken genome is an ongoing project and a new version should be released within the next few years. It is reasonable to believe that continuing efforts will contribute to elucidate the full sequence of the chicken genome in a near future. Until then, applying the dual transcript-discovery approach described here prior to the analysis of RNA-seq data per se enhances the sensitivity of gene expression profiles. This is particularly relevant considering that genes and splicing variants are specifically expressed in certain cell types or tissues, at different developmental stages and conditions within a single organism. For instance, we used the gene annotation model presented here as guide in a recent study, where we aimed at identifying genes that were regulated upon overexpression of connective tissue-associated transcription factors in chick micromass cultures (M.O., D.D., S.S., unpublished). More broadly, this approach could be also employed to analyse RNA-seq data of other organisms lacking manually-curated, high-quality reference annotation.

## MATERIALS AND METHODS

A complete description of tools, command lines, parameters and database links used for this study is provided as Supplementary Methods. The gene annotation model and Python scripts are accessible via SourceForge: https://sourceforge.net/projects/dualtranscriptdiscovery/.

### Chick embryos

Fertilized chick eggs were obtained from VALO BioMedia (Lohmann Selected Leghorn strain, Osterholz-Scharmbeck, Germany). Chick embryos were staged according to the number of days *in ovo* at 37.5°C.

### Chick micromass cultures

Two independent biological replicates of micromass cultures were prepared from limb buds of embryonic day (E) 4.5 chick embryos, infected with RCAS-BP(A) retroviruses carrying no recombinant protein and cultivated for 5 days as described previously (Solursh et al., 1978; Ibrahim et al., 2013). Briefly, ectoderm was dissociated by using a Dispase solution (Gibco) at 3 mg/ml and limb mesenchyme was digested by using a solution composed of 0.1% Collagenase type Ia (Sigma-Aldrich), 0.1% Trypsin (Gibco) and 5% FBS (Biochrom, Berlin, Germany) in DPBS (Gibco). Prior to seeding, mesenchymal cells were mixed with retroviruses (1:1) and maintained in culture for 5 days at 37°C in DMEM/Ham's F-12 (1:1) medium (Biochrom) supplemented with 10% FBS, 0.2% chicken serum (Sigma-Aldrich), 1% L-glutamine (Lonza, Basel, Switzerland) and 1% penicillin/streptomycin (Lonza).

### RNA sequencing

For both replicates, RNA extracts were obtained by harvesting 6 micromass cultures with RLT buffer (Qiagen). Total RNAs were purified by using the RNeasy mini kit (Qiagen) in combination to a DNase I (Qiagen) treatment to prevent genomic DNA contamination. RNA libraries were prepared by using the TruSeq Stranded mRNA Library Preparation kit (Illumina, San Diego, CA, USA), which enables the RNA strand orientation to be preserved. Strand-specific 50-bp paired-end reads were generated by using a HiSeq 2500 sequencer (Illumina) with a mean insert size of 150 bp.

### Genome-guided transcript discovery

RNA-seq data obtained from both biological replicates of micromass cultures were processed independently. Strand-specific read pairs were mapped against the chicken genome galGal4 (Hillier et al., 2004) by using TopHat2 v0.14 (Kim et al., 2013) (parameters: -r 150; -N 3; –read-edit-dist 3; –library-type fr-firststrand; -i 50; -G). UCSC (galGal4) and Ensembl (release 75) annotations were downloaded from Illumina iGenomes (http://support.illumina.com/sequencing/sequencing_software/igenome.html) and compared by using Cuffcompare from the Cufflinks suite v2.1.1 (Trapnell et al., 2010). Identical genes were retrieved only once and merged with the unique genes from each annotation. In case of discordant genes, the gene annotation with the best coverage was selected. The resulting gene annotation model composed of 17,318 genes was used as input for TopHat2 mapping. Transcript discovery was performed for each replicate by using Cufflinks v2.1.1 (Trapnell et al., 2010) (parameters: -b; -u; -library-type, fr-firststrand; -g) and the combined gene annotation model as guide. Resulting annotations were merged into a single model by using the Cufflinks tool Cuffmerge v2.1.1 (Trapnell et al., 2010).

### *De novo* transcript discovery

A second transcript-discovery approach was led independently of the genome sequence. Low-quality RNA-seq reads from each replicate of micromass cultures were first filtered out by using the FASTX-Toolkit v0.0.13 (http://hannonlab.cshl.edu/fastx_toolkit/). Reads with a median quality value lower than 28 were discarded. Filtered read pairs were then trimmed by using Trimmomatic v0.32 (Bolger et al., 2014) (parameters: ILLUMINACLIP TruSeq3 paired-end for HiSeq, seedMismatches 2, palindromeClipThreshold 30, simpleClipThreshold 10; LEADING 5; TRAILING 5; MINLEN 36). Complete read pairs were then assembled by using Trinity r20140717 (Grabherr et al., 2011) (default parameters except for the strand-specific library orientation set at RF).

### Gene fragmentation correction

Contigs resulting from the *de novo* assembly were compared to the gene candidate sequences obtained by the first approach by using BLASTN from BLAST+ v2.2.31+ (Camacho et al., 2009) (parameters: -strand plus; -dust no; -soft_masking no). Contigs were assigned to a given gene candidate if they matched at least 40 bp that were not covered by a previous hit with a percentage of identities higher than 90%. Assigned contigs that were not fully covered by a given gene candidate were further processed to extract continuous uncovered regions of at least 400 bp. Remaining contigs were mapped against the galGal4 genome by using BLASTN (parameters: -perc_identity 90; -dust no; -soft_masking no). Contigs were assigned to a given gene candidate if they were located between two gene features, potentially corresponding to an exon missed by Cufflinks, or in the vicinity of a first or last exon, potentially corresponding to a missing 5′- or 3′-untranslated region (UTR), respectively. Remaining unmapped contigs were retrieved as they could correspond to non-defined genomic regions. Unmapped, unassigned and non-covered contigs or regions of at least 400 bp were further processed to remove redundant sequences between multiple isoforms. This step was necessary to prevent read pairs to be mapped on multiple gene features and to be consequently discarded during fragment counting. Isoforms belonging to the same gene candidates defined by Trinity were compared to the longest isoforms by using BLASTN (parameters: -perc_identity 90; -strand plus; -dust no; -soft_masking no; -ungapped). Sequence alignments were then examined to build consensus gene sequences by merging identical sequences between two isoforms and by adding sequences unique to each isoform. Pairwise sequence comparison was performed until all isoforms of the same gene candidates were processed and concatenated. Resulting contig sequences were gathered together as an artificial chromosome and separated to each other by 250 bp of nucleotides N, corresponding to the total length of read pairs (50 bp for each read and 150 bp as insert size).

### Functional annotation

Gene candidate sequences retrieved from both transcript-discovery approaches were then compared to existing databases for gene name assignment. First, gene candidates were compared to the NCBI RefSeq transcript database by using BLASTN (parameters: -strand plus; -dust no; -soft_masking no). Comparison was limited to Aves (birds) sequences (taxid 8782). Gene candidates with a percentage of identities >90% for chicken genes or 75% for bird genes, and bidirectionally covered on at least 50% of their length were assigned to the corresponding hits. Gene candidates matching several discordant gene names, such as chimeric and fused gene candidates, were manually investigated and corrected. Non-annotated gene candidate sequences were then compared to the NCBI human (taxid 9606) and mouse (taxid 10090) non-redundant protein database by using BLASTX from BLAST+ v2.2.31+ (Camacho et al., 2009) (parameters: -strand, plus; -seg, no). Gene candidates with a percentage of homology of at least 30% and covered by at least 50% of their length were filtered. Matching protein accession numbers were converted into gene accession numbers by using the Hyperlink Management System (Imanishi and Nakaoka, 2009). ORF prediction was finally performed on remaining gene candidates by using TransDecoder v2.1.0 (Haas et al., 2013) (strand specificity parameter: -S). ORFs of at least 100 amino acids were annotated by using Trinotate v3.0.1 (https://trinotate.github.io/). Functional annotation was based on the following protein predictions: (i) BLASTX and BLASTP homology search against the SwissProt database (Bairoch et al., 2004); (ii) protein domain prediction against the Pfam database (Punta et al., 2012) by using HMMER v3.1b2 (Finn et al., 2011); (iii) signal peptide prediction by using SignalP v4.1 (Petersen et al., 2011); and (iv) transmembrane domain prediction by using tmHMM v2.0c (Krogh et al., 2001). Resulting functional annotation was divided into three categories: (i) putative proteins, for which at least one protein domain could be identified; (ii) uncharacterized proteins, corresponding to ORFs for which no protein domain could be identified; and (iii) ncRNAs, corresponding to genes with an ORF shorter than 100 amino acids.

### Comparison with galGal5

UCSC (galGal5) and Ensembl (release 89) reference annotations associated with the galGal5 genome version were downloaded from the UCSC browser and merged by using the Cufflinks tool Cuffmerge v2.1.1 (Trapnell et al., 2010). RNA-seq strand-specific read pairs were mapped against the chicken genome galGal5 (Warren et al., 2017) by using TopHat2 v0.14 (Kim et al., 2013) (parameters: -r 150; -N 3; –read-edit-dist 3; –library-type fr-firststrand; -i 50; -G) and the merged reference annotations as guide. Sequences of annotated galGal5 transcripts were retrieved from the RefSeq database (ftp://ftp.ncbi.nih.gov/genomes/Gallus_gallus/RNA/) and compared to the predicted gene candidates by using BLASTN (parameters: -perc_identity 90; -strand plus; -dust no; -soft_masking no). On one hand, the total length coverage of predicted gene candidates was assessed by identifying all regions matching with galGal5 gene sequences. On the other hand, gene name assignment between predicted gene candidates and annotated galGal5 genes was compared by retrieving only the hits that matched at least 50% of their length.

### Fragment counting

Strand-specific read pairs mapped against the chicken genome and the artificial chromosome generated from the *de novo* transcript discovery were first split by strand by using SAMtools v1.2 (Li et al., 2009) according to their FLAG field (strand plus: -f 128 -F 16, -f 80; strand minus: -f 144, -f 64 -F 16). Fragments (both reads of a pair) mapped on gene features were counted by using featureCounts v1.4.6-p3 (Liao et al., 2014) (parameters: -p; -s 2; –ignoreDup; -B; -R). Chimeric fragments aligned on different chromosomes were taken into consideration to overcome the gene fragmentation due to the location of gene parts on multiple chromosome contigs.

## Supplementary Material

Supplementary information
