# Supplementary material for: A dual transcript-discovery approach to improve the delimitation of gene features from RNA-seq data in the chicken model
Source: Biol Open. 2017 Nov 28;7(1):bio028498. doi: 10.1242/bio.028498 (PMC5827264; doi:10.1242/bio.028498)
Supplement: Supplementary information [file biolopen-7-028498-s1.pdf]

A dual transcript-discovery approach to improve the delimitation of gene features from RNA-seq data in the chicken model: Supplementary Methods

1) Requirements:

- TopHat2 v0.14
- Cufflinks v2.1.1
- FASTX-toolkit v0.0.13
- Trimmomatic v0.32
- Trinity r20140717
- BLAST+ v2.2.31+
- BEDtools v2.24.0
- TransDecoder v2.1.0
- HMMER v3.1b2
- SignalP v4.1
- tmHMM v2.0c
- Trinotate v3.0.1
- Python v2.7 (scripts are available at: https://dualtranscriptdiscovery.sourceforge.io/)

2) Datasets:

Strand-specific paired-end reads (length of 50 bp, mean insert size of 150 bp) were generated by using a HiSeq 2500 sequencer (Illumina). Datasets used for this study are available at:
https://www.ncbi.nlm.nih.gov/geo/query/acc.cgi?acc=GSM2685833
https://www.ncbi.nlm.nih.gov/geo/query/acc.cgi?acc=GSM2685834
The reference sequence of the chicken galGal4 genome can be downloaded from the UCSC browser via the following link: http://hgdownload.soe.ucsc.edu/goldenPath/galGal4/bigZips/galGal4.fa.gz.
Length of each chromosome and contig associated with the chicken gaGal4 genome are available at: http://hgdownload.soe.ucsc.edu/goldenPath/galGal4/bigZips/galGal4.chrom.sizes.
Ensembl and UCSC gene annotation models related to galGal4 are accessible via Illumina iGenomes: https://support.illumina.com/sequencing/sequencing_software/igenome.html.

3) Genome-guided transcript discovery:

3.1) Read mapping: TopHat2

With a reference gene annotation model

```
$ tophat -r 150 -N 3 --read-edit-dist 3 --library-type fr-firststrand -i 50 \
    -G genes.gtf genome reads_R1.fq.gz reads_R2.fq.gz
```

Without a reference gene annotation model

```
$ tophat -r 150 -N 3 --read-edit-dist 3 --library-type fr-firststrand -i 50 \
    genome reads_R1.fq.gz reads_R2.fq.gz
```

3.2) Gene prediction: Cufflinks

With a reference gene annotation model

```
$ cufflinks -b genome.fa -u -library-type fr-firststrand -g genes.gtf \
    accepted_hits.bam
```

Without a reference gene annotation model

```
$ cufflinks -b genome.fa -u -library-type fr-firststrand accepted_hits.bam
```

3.3) Merge gene annotation models: Cuffmerge

Create a file listing the name of the gene annotation models generated for each replicate

```
./transcripts_Rep1.gtf
./transcripts_Rep2.gtf
```


Biology Open • Supplementary information

Create a single gene annotation model

```
$ cuffmerge list_models.txt
```

4) *De novo* transcript discovery:

4.1) Merge reads from both replicates

```
$ cat reads_R1_Rep1.fq reads_R1_Rep2.fq > reads_R1.fq
$ cat reads_R2_Rep1.fq reads_R2_Rep2.fq > reads_R2.fq
```

4.2) Filter reads by quality: FASTX-Toolkit

```
$ fastq_quality_filter -q 28 -p 50 -i reads_R1.fq -o reads_R1.filtered.fq
$ fastq_quality_filter -q 28 -p 50 -i reads_R2.fq -o reads_R2.filtered.fq
```

4.3) Trim reads by quality: Trimmomatic

```
$ java -jar trimmomatic-0.32.jar PE reads_R1.filtered.fq reads_R2.filtered. \
     reads_R1.trimmed.fq reads_R1.unpaired.fq \
     reads_R2.trimmed.fq reads_R2.unpaired.fq \
     ILLUMINACLIP:TruSeq3-PE:2:30:10 LEADING:5 TRAILING:5 MINLEN:36
```

4.4) *De novo* assembly: Trinity

```
$ $TRINITY_HOME/Trinity.pl --seqType fq --JM 10G --SS_lib_type RF \
     --left reads_R1.trimmed.fq --right reads_R2.trimmed.fq
```

5) Gene fragmentation correction

5.1) Retrieve transcript sequences

Upload the gene annotation model “merged.gtf” resulting from the genome-guided transcript discovery on the UCSC browser. Transcript sequences can be retrieved by using the Table Browser tool.

5.2) Create a BLAST database: BLAST+

```
$ makeblastdb -in transcripts.fa -dbtype nucl
```

5.3) Compare Trinity contigs to transcripts: BLAST+

```
$ blastn -query contigs.fa -db transcripts.fa -perc_identity 90 \
     -strand plus -dust no -soft_masking no -outfmt "7 std qlen slen sstrand" \
     -out contigs_vs_transcripts.blastn
```

5.4) Convert transcript IDs into gene IDs in the BLAST output file: Python script

```
$ python convert_tids_into_gids.py \
     merged.gtf contigs_vs_transcripts.blastn contigs_vs_genes.blastn
```

5.5) Assign Trinity contigs to genes: Python script

The minimum number of overlapping base pairs not covered from previous hits can be fixed by adjusting the parameter “t_aln_length=40”.

```
$ python assign_contigs_to_genes.py \
     contigs_vs_genes.blastn 40 \
     assigned_contigs.txt
```


Biology Open • Supplementary information

5.6) Extract unassigned Trinity contigs: Python script

```
$ python extract_unassigned_contigs.py \
    contigs.fa assigned_contigs.txt unassigned_contigs.fa
```

5.7) Create a BLAST database: BLAST+

```
$ makeblastdb -in genome.fa -dbtype nucl
```

5.8) Compare unassigned Trinity contigs to genome: BLAST+

```
$ blastn -query unassigned_contigs.fa -db genome.fa -perc_identity 90 \
    -dust no -soft_masking no -outfmt "7 std qlen slen sstrand" \
    -out unassigned_contigs_vs_genome.blastn
```

5.9) Filter hits based on cumulative alignment length: Python script

The minimum number of overlapping base pairs not covered from previous hits can be fixed by adjusting the parameter "t_aln_length=40". The minimum percentage of cumulative alignment length can be fixed by adjusting the parameter "p_cumul_length=50".

```
$ python parse_blast_hits_genome.py \
    unassigned_contigs_vs_genome.blastn 40 50 \
    unassigned_contigs_vs_genome.blastn.txt
```

5.10) Extract genome coordinates from filtered hits: Python script

```
$ python extract_genome_coordinates.py \
    unassigned_contigs_vs_genome.blastn.txt \
    unassigned_contigs_vs_genome.blastn.bed
```

5.11) Sort genome coordinates: sort

```
$ sort -k1,1 -k2,2n unassigned_contigs_vs_genome.blastn.bed \
    > unassigned_contigs_vs_genome.blastn.sort.bed
```

5.12) Extract gene coordinates: Python script

```
$ python extract_gene_coordinates.py \
    merged.gtf genes.bed
```

5.13) Extend gene boundaries by 1000 bp: BEDtools

```
$ bedtools slop -i genes.bed -g galGal4.chrom.sizes -b 1000 \
    > genes.extended.bed
```

5.14) Sort gene coordinates: sort

```
$ sort -k1,1 -k2,2n genes.extended.bed > genes.extended.sort.bed
```

5.15) Compare unassigned Trinity contigs to genes: BEDtools

```
$ bedtools intersect -a unassigned_contigs_vs_genome.blastn.sort.bed \
    -b genes.extended.bed -wo -s > unassigned_contigs_vs_genes.bed
```

5.16) Assign Trinity contigs to genes: Python script

```
$ python assign_unassigned_contigs_to_genes.py \
    unassigned_contigs_vs_genes.bed \
    unassigned_contigs.txt
```

5.17) Extract unmapped Trinity contigs: Python script

```
$ python extract_unmapped_contigs.py \
    contigs.fa assigned_contigs.txt unassigned_contigs.txt \
    unmapped_contigs.txt
```


Biology Open • Supplementary information

5.18) Select regions of assigned, unassigned and unmapped Trinity contigs: Python script

The minimum number of continuous base pairs not covered can be fixed by adjusting the parameter "min_length=400".

```
$ python select_contig_regions.py \
    contigs.fa assigned_contigs.txt \
    unassigned_contigs.txt unmapped_contigs.txt 400 \
    contig_regions.fa
```

6) Remove redundant sequences from selected Trinity contigs

6.1) Extract Trinity contigs with multiple isoforms: Python script

```
$ python extract_gene_isoforms.py \
    contig_regions.fa \
    contigs_with_single_isoform.fa \
    contigs_with_multiple_isoforms.fa
```

6.2) Extract longest isoforms: Python script

```
$ python extract_longest_isoforms.py \
    contigs_with_multiple_isoforms.fa \
    0_contigs_longest_isoforms.fa \
    0_contigs_longest_isoforms.index
```

6.3) Create a BLAST database: BLAST+

```
$ makeblastdb -in 0_contigs_longest_isoforms.fa -dbtype nucl
```

6.4) Compare Trinity contigs with multiple isoforms to longest isoforms: BLAST+

```
$ blastn -query contigs_with_multiple_isoforms.fa \
    -db 0_contigs_longest_isoforms.fa \
    -perc_identity 90 -strand plus -dust no -soft_masking no -ungapped \
    -outfmt "7 std qlen slen sstrand" \
    -out 0_contigs_with_multiple_isoforms_vs_longest_isoforms.blastn
```

6.5) Parse BLAST hits to build contig scaffolds: Python script

```
$ python build_scaffolds.py \
    0_contigs_longest_isoforms.index \
    0_contigs_with_multiple_isoforms_vs_longest_isoforms.blastn \
    0_contigs_longest_isoforms.scaffolds
```

6.6) Build contig sequences based on scaffolds: Python script

```
$ python build_consensus_sequences.py \
    contigs_with_multiple_isoforms.fa \
    0_contigs_longest_isoforms.scaffolds \
    0_contigs_longest_isoforms.fa 0_contigs_longest_isoforms.index \
    1_contigs_longest_isoforms.fa 1_contigs_longest_isoforms.index
```

6.7) Incremental Trinity contig concatenation: Python script

The four previous steps are executed to include sequences that were not processed before until all contig sequences are concatenated.

```
$ python concatenate_gene_sequences.py \
    contigs_with_multiple_isoforms.fa \
    contigs_with_multiple_isoforms_vs_longest_isoforms.blastn \
    contigs_longest_isoforms
```

7) Functional annotation: birds

7.1) Download the latest release of the NCBI nucleotide sequence database

```
$ wget ftp://ftp.ncbi.nlm.nih.gov/blast/db/FASTA/nt.gz
```

7.2) Retrieve taxonomy data to build the custom database

Download taxonomy IDs related to the class Aves (birds, taxid 8782):
https://www.ncbi.nlm.nih.gov/taxonomy/?term=txid8782[Subtree].

Download taxid mapping for nucleotide sequence records:
ftp://ftp.ncbi.nih.gov/pub/taxonomy/accession2taxid/nucl_gb.accession2taxid.gz.

Download taxonomy information for the database:
ftp://ftp.ncbi.nlm.nih.gov/blast/db/taxdb.tar.gz.

7.3) Create taxid map of Birds accession IDs: Python script

```
$ python create_taxid_map.py \
    Birds_taxids.txt nucl_gb.accession2taxid \
    Birds_taxid_map.txt
```

7.4) Parse Birds sequences from NCBI nt database: Python script

```
$ python parse_db_seqids.py \
    Birds_taxid_map.txt nt \
    nt_Birds
```

7.5) Create a BLAST database: BLAST+

```
$ makeblastdb -in nt_Birds -dbtype nucl \
    -taxid_map Birds_taxid_map.txt -parse_seqids -hash_index
```

7.6) Merge all transcripts and contigs

```
$ cat transcripts.fa contigs_with_single_isoform.fa \
    contigs_longest_isoforms.fa > all_gene_candidates.fa
```

7.7) Compare transcripts and contigs with Birds nucleotide sequences: BLAST+

```
$ blastn -query all_gene_candidates.fa -db nt_Birds \
    -perc_identity 75 -strand plus -dust no -soft_masking no \
    -outfmt "7 std qlen slen sstrand sallseqid salltitles staxids sscinames" \
    -out all_gene_candidates_vs_Birds.blastn
```

7.8) Parse BLAST hits for Birds gene assignment: Python script

The minimum percentages of identities for chicken genes and for other bird genes can be fixed by adjusting the parameters "ggal_pcid=90" and "other_pcid=75", respectively. The minimum percentages of matching cumulative length for the query and for the subject can be fixed by adjusting the parameters "q_clen=50" and "s_clen=50", respectively.

```
$ python parse_blast_hits_birds.py \
    all_gene_candidates_vs_Birds.blastn 90 75 50 50 \
    all_gene_candidates_vs_Birds.hits.txt
```

7.9) Retrieve gene information from the NCBI RefSeq database:

Download RefSeq gene report: ftp://ftp.ncbi.nlm.nih.gov/gene/DATA/gene2accession.gz.

Biology Open • Supplementary information

7.10) Convert Birds nucleotide accession IDs into gene symbols: Python script

```
$ python get_gene_symbols.py \
    Birds_taxids.txt gene2accession nucl \
    all_gene_candidates_vs_Birds.hits.txt \
    all_gene_candidates_vs_Birds.assignment.txt
```

8) Functional annotation: human and mouse

8.1) Download the latest release of the NCBI protein sequence database

```
$ wget ftp://ftp.ncbi.nlm.nih.gov/blast/db/FASTA/nr.gz
```

8.2) Retrieve taxonomy data to build the custom database

Create a file listing the taxonomy IDs related to *Homo sapiens* (9606) and *Mus musculus* (10090) species.

Download taxid mapping for protein sequence records:
ftp://ftp.ncbi.nih.gov/pub/taxonomy/accession2taxid/prot.accession2taxid.gz

8.3) Create taxid map of human/mouse accession IDs: Python script

```
$ python create_taxid_map.py \
    Mammals_taxids.txt prot.accession2taxid \
    Mammals_taxid_map.txt
```

8.4) Parse human/mouse sequences from NCBI nr database: Python script

```
$ python parse_db_seqids.py \
    Mammals_taxid_map.txt nr \
    nr_Mammals
```

8.5) Create a BLAST database: BLAST+

```
$ makeblastdb -in nr_Mammals -dbtype prot \
    -taxid_map Mammals_taxid_map.txt -parse_seqids -hash_index
```

8.6) Extract unannotated transcripts and contigs from comparison with Birds: Python script

```
$ python extract_nonannotated_genes.py \
    all_gene_candidates_vs_Birds.assignment.txt \
    all_gene_candidates.fa \
    Birds_nonannotated_gene_candidates.fa
```

8.7) Compare transcripts and contigs with human/mouse protein sequences: BLAST+

```
$ blastx -query Birds_nonannotated_gene_candidates.fa \
    -db nr_Mammals -strand plus -seg no \
    -outfmt "7 std qlen slen qframe sallseqid salltitles staxids sscinames" \
    -out Birds_nonannotated_gene_candidates_vs_Mammals.blastx
```

8.8) Parse BLAST hits for Mammals gene assignment: Python script

The minimum percentage of identities for human/mouse proteins can be fixed by adjusting the parameters "mam_pcid=30". The minimum percentage of matching cumulative length for the query can be fixed by adjusting the parameters "q_clen=50".

```
$ python parse_blast_hits_mammals.py \
    Birds_nonannotated_gene_candidates_vs_Mammals.blastx 30 50 \
    Birds_nonannotated_gene_candidates_vs_Mammals.hits.txt
```


Biology Open • Supplementary information

8.9) Convert Mammals protein accession IDs into gene symbols: Python script

```
$ python get_gene_symbols.py \
    Mammals_taxids.txt gene2accession prot \
    Birds_nonannotated_gene_candidates_vs_Mammals.hits.txt \
    Birds_nonannotated_gene_candidates_vs_Mammals.assignment.txt
```

9) Functional annotation: ORF and protein domain prediction

Remaining unassigned transcripts and contigs were annotated according to the Trinotate pipeline. Procedure including tools and database links is described at: http://trinotate.github.io/.

9.1) Extract unannotated transcripts and contigs from comparison with Mammals: Python script

```
$ python extract_nonannotated_genes.py \
    Birds_nonannotated_gene_candidates_vs_Mammals.assignment.txt \
    Birds_nonannotated_gene_candidates.fa \
    Mammals_nonannotated_gene_candidates.fa
```

9.2) ORF prediction: TransDecoder

```
$ TransDecoder.LongOrfs -t Mammals_nonannotated_gene_candidates.fa -S
```

9.3) Create the BLAST UniProt database: BLAST+

```
$ makeblastdb -in uniprot_sprot.pep -dbtype prot
```

9.4) Compare transcripts and contigs to UniProt database: BLAST+

```
$ blastx -query Mammals_nonannotated_gene_candidates.fa \
    -db uniprot_sprot.pep -strand plus -seg no \
    -max_target_seqs 1 -outfmt 6 \
    -out Mammals_nonannotated_gene_candidates_vs_UniProt.blastx
```

9.5) Compare predicted ORFs to UniProt database: BLAST+

```
$ blastp -query TransDecoder_predicted_ORFs.pep \
    -db uniprot_sprot.pep -seg no -max_target_seqs 1 -outfmt 6 \
    -out TransDecoder_predicted_ORFs_vs_UniProt.blastp
```

9.6) Create the HMMER Pfam database: HMMER

```
$ hmmpress Pfam-A.hmm
```

9.7) Pfam protein domain prediction: HMMER

```
$ hmmscan --domtblout TransDecoder_predicted_ORFs_vs_Pfam.out \
    Pfam-A.hmm TransDecoder_predicted_ORFs.pep \
    > TransDecoder_predicted_ORFs_vs_Pfam.log
```

9.8) Signal peptide prediction: SignalP

```
$ signalp -f short -n TransDecoder_predicted_ORFs_vs_SignalP.out \
    TransDecoder_predicted_ORFs.pep \
    > TransDecoder_predicted_ORFs_vs_SignalP.log
```

9.9) Transmembrane domain prediction: tmHMM

```
$ tmhmm --short TransDecoder_predicted_ORFs.pep \
    TransDecoder_predicted_ORFs_vs_tmHMM.out
```


Biology Open • Supplementary information

9.10) Create gene-to-transcript mapping file: Python script

```
$ python create_gene_transcript_map.py \
    Mammals_nonannotated_gene_candidates.fa \
    Mammals_nonannotated_gene_candidates_transcript.map
```

9.11) Functional annotation and analysis: Trinotate

```
# Initialize database
$ Trinotate Trinotate.sqlite init \
    --gene_trans_map Mammals_nonannotated_gene_candidates_transcript.map \
    --transcript_fasta Mammals_nonannotated_gene_candidates.fa \
    --transdecoder_pep TransDecoder_predicted_ORFs.pep
# Load BLASTX transcript hits
$ Trinotate Trinotate.sqlite \
    LOAD_swissprot_blastx
    Mammals_nonannotated_gene_candidates_vs_UniProt.blastx
# Load BLASTP protein hits
$ Trinotate Trinotate.sqlite \
    LOAD_swissprot_blastp TransDecoder_predicted_ORFs_vs_UniProt.blastp
# Load Pfam protein domain prediction
$ Trinotate Trinotate.sqlite \
    LOAD_pfam TransDecoder_predicted_ORFs_vs_Pfam.out
# Load SignalP signal peptide prediction
$ Trinotate Trinotate.sqlite \
    LOAD_signalp TransDecoder_predicted_ORFs_vs_SignalP.out
# Load tmHMM transmembrane domain prediction
$ Trinotate Trinotate.sqlite \
    LOAD_tmhmm TransDecoder_predicted_ORFs_vs_tmHMM.out
# Export Trinotate annotation report
$ Trinotate Trinotate.sqlite report > Trinotate_report.xls
```
